# Multiple ESBL-Producing *Escherichia coli* Sequence Types Carrying Quinolone and Aminoglycoside Resistance Genes Circulating in Companion and Domestic Farm Animals in Mwanza, Tanzania, Harbor Commonly Occurring Plasmids

**DOI:** 10.3389/fmicb.2016.00142

**Published:** 2016-02-11

**Authors:** Jeremiah Seni, Linda Falgenhauer, Nabina Simeo, Mariam M. Mirambo, Can Imirzalioglu, Mecky Matee, Mark Rweyemamu, Trinad Chakraborty, Stephen E. Mshana

**Affiliations:** ^1^Department of Microbiology and Immunology, Catholic University of Health and Allied SciencesMwanza, Tanzania; ^2^Institute of Medical Microbiology, Justus-Liebig UniversityGiessen, Germany; ^3^German Center for Infection Research, DZIF Partner Site Giessen-Marburg-LangenGiessen, Germany; ^4^Department of Microbiology/Immunology, Muhimbili University of Health and Allied Sciences Dar es Salaam, Tanzania; ^5^Southern African Centre for Infectious Disease Surveillance, Sokoine University of AgricultureMorogoro, Tanzania

**Keywords:** farming communities, companion animals, domestic farm animals, ESBL, Mwanza, Tanzania

## Abstract

The increased presence of extended-spectrum beta-lactamase (ESBL)-producing bacteria in humans, animals, and their surrounding environments is of global concern. Currently there is limited information on ESBL presence in rural farming communities worldwide. We performed a cross-sectional study in Mwanza, Tanzania, involving 600 companion and domestic farm animals between August/September 2014. Rectal swab/cloaca specimens were processed to identify ESBL-producing Enterobacteriaceae. We detected 130 (21.7%) animals carrying ESBL-producing bacteria, the highest carriage being among dogs and pigs [39.2% (51/130) and 33.1% (43/130), respectively]. The majority of isolates were *Escherichia coli* [93.3% (125/134)] and exotic breed type [OR (95%CI) = 2.372 (1.460–3.854), p-value < 0.001] was found to be a predictor of ESBL carriage among animals. Whole-genome sequences of 25 ESBL-producing *E. coli* were analyzed for phylogenetic relationships using multi-locus sequence typing (MLST) and core genome comparisons. Fourteen different sequence types were detected of which ST617 (7/25), ST2852 (3/25), ST1303 (3/25) were the most abundant. All isolates harbored the *bla*_CTX-M-15_ allele, 22/25 carried *strA* and *strB*, 12/25 *aac(6′)-lb-cr*, and 11/25 *qnrS1*. Antibiotic resistance was associated with IncF, IncY, as well as non-typable plasmids. Eleven isolates carried pPGRT46-related plasmids, previously reported from isolates in Nigeria. Five isolates had plasmids exhibiting 85–99% homology to pCA28, previously detected in isolates from the US. Our findings indicate a pan-species distribution of ESBL-producing *E. coli* clonal groups in farming communities and provide evidence for plasmids harboring antibiotic resistances of regional and international impact.

## Introduction

Extended-spectrum beta-lactamases (ESBL) are enzymes encoded on the chromosome or on plasmids, conferring resistance to penicillins, cephalosporins, and monobactams (Bradford, 2001). The burden of ESBL is currently of global concern not only to humans but also animals and the ecosystem at large (Hawkey and Jones, 2009; Dierikx et al., 2012; Geser et al., 2012; Lupindu et al., 2014). This is due to interfaces that promote and enable the transmission of strains and antibiotic resistance genes harbored therein (Lupindu et al., 2014; Rubin and Pitout, 2014). Different factors for ESBL fecal carriage have been reported in both humans and animals, such as previous exposure to antimicrobial agents, hygienic behavior, or microbial inherent genetic factors (Bester and Essack, 2008; Geser et al., 2012; Lupindu et al., 2014; Nelson et al., 2014). The prevailing evidence from hospital and community-based studies in Mwanza, Tanzania, suggests that CTX-M-15 is predominant in ESBL-producing clinical isolates and is associated with significant morbidity and mortality (Kayange et al., 2010; Mshana et al., 2011a). Moreover, despite evidence from other regions in Tanzania on the growing problem of antimicrobial resistance in animals associated with varying ESBL-alleles (Katakweba, 2014; Lupindu et al., 2014), there is still limited information regarding genetic diversity of ESBL isolates among companion and domestic farm animals in the Mwanza region. Therefore, this study aimed to address the magnitude and the risk factors associated with ESBL carriage, identify ESBL alleles and analyze the MLST types among companion and domestic farm animals in this region.

## Materials and Methods

### Study Design, Site, and Sampling Procedures

This cross-sectional study was conducted between August and September 2014, and involved 600 companion and domestic farm animals in three districts of the Mwanza region (Ilemela, Nyamagana, and Misungwi). Healthy dogs, sheep, goats, chickens, pigs, and cattle were sampled. In case of a herd of animals, 10% of animals were randomly selected and sampled.

### Data Collection and Laboratory Procedures

Demographic data such as sex, residence, breed type, history of antibiotic use, and type of antibiotics used were collected. Rectal swabs from pigs, cattle, sheep, goats, and dogs and cloaca swabs from layers and local chickens were collected and placed in Amies transport medium (MAST GROUP Ltd., Bootle, UK). All samples were taken to the Catholic University of Health and Allied Sciences (CUHAS) laboratory for microbiological analysis.

### Detection of ESBLs

Each fecal sample was plated on MacConkey agar (HI Media, Mumbai, India) supplemented with cefotaxime (2 μg/mL) and incubated at 35–37°C for 24–48 h. Predominant colonies were further identified using in-house biochemical tests as previously described (Koneman et al., 1997). Pure colonies were grown on ESBL ChromAgar (MAST GROUP Ltd.) for ESBL confirmation and further characterization based on different color of the colonies (*Escherichia coli*: dark pinkish to reddish colonies; *Klebsiella* spp./*Enterobacter* spp: metallic blue; *Proteus* spp: brown halo colonies).

### Antimicrobial Susceptibility Testing

Drug susceptibility testing was done by Kirby–Bauer disk diffusion method on Mueller–Hinton agar (HI Media) based on recommendations of the Clinical Laboratory Standard Institute (Clinical Laboratory Standards Institute [CLSI], 2011). Resistance to the non-beta-lactam antimicrobials ciprofloxacin (5 μg), gentamicin (10 μg), tetracycline (30 μg) and trimethoprim/sulphamethoxazole (1.25/23.75 μg) was tested. For quality control *E. coli* ATCC 25922 and *E. coli* ATCC 35218 were used.

### Data Management and Analysis

Demographic data and laboratory results were entered into a log book, sorted, and transferred to Microsoft Excel. Analysis was done using STATA version 11.0 (STATA, College Station, TX, USA). Results were presented into percentages/proportions for categorical variables and median (IQR) for continuous variables. Univariate and multivariate logistic regression analyses were performed to identify independent predictors of ESBL carriage among the investigated animals. A *p*-value of < 0.05 was considered to be a statistical significant cut-off.

### Whole-Genome Sequencing and Phylogenetic Analysis

Twenty-five ESBL-producing *E. coli* isolates were chosen for whole-genome sequencing (WGS). The isolates were confirmed to be ESBL-producers using the VITEK®2 compact system (bioMérieux, Nürtingen, Germany). DNA was isolated using the Purelink Genome DNA Mini kit (Invitrogen, Darmstadt, Germany). WGS was carried out on an Illumina MiSeq instrument (Illumina, San Diego, CA, USA) using an Illumina Nextera XT library with 2x300-bp paired-end reads. The data was assembled using SPAdes (version 3.0) (Bankevich et al., 2012). Contigs larger than 500-bp and a coverage higher than nine were ordered to *E. coli* MG1655 (accession number U00096.3) using MAUVE (Darling et al., 2004). The contigs were concatenated to generate pseudogenomes. Whole-genome phylogeny was determined by the software package Harvest Suite using *E. coli* MG1655 as reference (Treangen et al., 2014). The phylogenetic tree was drawn using MEGA5 (Hall, 2013).

The raw data of the sequenced *E. coli* are available at the European Nucleotide Archive (ENA), under the project number PRJEB12335.

### *In Silico* Analyses

Sequences were analyzed for MLST (according to Wirth et al., 2006), transferrable resistance genes, plasmid replicon types and pMLST using MLST 1.8, ResFinder, Plasmidfinder and pMLST software of the Center for Genomic epidemiology (Larsen et al., 2012; Zankari et al., 2012; Carattoli et al., 2014). Phylogenetic groups were determined as previously described (Clermont et al., 2000). The presence of *chu*A, *yja*A and TSPE4C.2 sequences was examined using blastN (Altschul et al., 1990). The location of *bla*_CTX-M-15_ was determined by analyzing the contigs harboring *bla*_CTX-M-15_ using blastN. Plasmid comparisons were carried out using the software BRIG (Alikhan et al., 2011).

### Study Approval and Ethical Considerations

Ethical clearance and approval to conduct this study was obtained from CUHAS/Bugando Medial Centre Ethics Review Board (CREC/043/2014). Permission to conduct the study was sought from Mwanza city authority. Voluntary and written informed consent was sought from every owner/keeper of the animals prior to inclusion in the study.

### Study Limitation

The findings from this study are based on companion and domestic farm animals in the selected districts in the Mwanza region and may not be generalized to companion and domestic farm animals throughout the country.

## Results

### Baseline Characteristics

During the study period a total of 600 animals were sampled with each animal type constituting approximately 16.7% (100/600). Of the 600 animals, the majority was of local breed [66.6% (400/600)] (**Supplementary Table S1**).

### ESBL Carriage Rates

The ESBL carriage rate among companion and domestic farm animals was 21.7% (130/600) [95% CI; 18.4–24.9] with the highest carriage among dogs and pigs [39.2% (51/130) and 33.1% (43/130), respectively] (**Supplementary Table S2**). Four animals had double carriage. Thereby, the total number of ESBL-producing isolates was 134 (**Table 1**). The majority of the ESBL isolates were *E. coli* [93.3% (125/134)], whereas *Klebsiella* spp. and *Proteus* spp. accounted for the remaining 6.0% (8/134) and 0.7% (1/134), respectively.

**Table 1:** Antimicrobial susceptibility profiles of ESBL isolates from companion and domestic farm animals.

Antimicrobial susceptibility		ESBL-producing bacteria (*n* = 134)		
		
		*Escherichia coli n* (%)	*Klebsiella* sp. *n* (%)	*Proteus* sp. n (%)
Trimethoprim/	Resistant	113 (90.4)	4 (50.0)	0 (0.0)
sulphamethoxazole	Intermediate	2 (1.6)	1 (12.5)	0 (0.0)
	Sensitive	10 (8.0)	3 (37.5)	1 (100.0)
Tetracycline	Resistant	79 (63.2)	3 (37.5)	0 (0.0)
	Intermediate	29 (23.2)	2 (25.0)	0 (0.0)
	Sensitive	17 (13.6)	4 (50.0	1 (100.0)
Ciprofloxacin	Resistant	42 (33.6)	5 (62.5)	0 (0.0)
	Intermediate	1 (0.1)	0 (0.0)	0 (0.0)
	Sensitive	82 (65.5)	3 (37.5)	1 (100.0)
Gentamicin	Resistant	24 (19.2)	4 (50.0)	0 (0.0)
	Intermediate	16 (12.8)	0 (0.0)	0 (0.0)
	Sensitive	85 (68.0)	4 (4.0)	1 (100.0)


### Antimicrobial Resistance of ESBL Isolates

The majority of *E. coli* ESBL-producing isolates were resistant to trimethoprim/sulphamethoxazole and tetracycline (90.4 and 63.2%, respectively). Resistance to ciprofloxacin and gentamicin accounted for 33.6 and 19.2%, respectively (**Table 1**). All *E. coli* isolates which underwent WGS exhibited a minimum inhibitory concentration for cefotaxime and ceftriaxone greater than 32 and 8 μg/mL, respectively.

### Association of ESBL Carriage with Variables

On bivariate analysis, male animals were likely to be more colonized with ESBL isolates as compared to females (30.5% vs. 18.1%, *p*-value = 0.001). Exotic breeds were more colonized with ESBL isolates as compared to local breeds (28.5% vs. 18.3%, *p*-value = 0.004). Moreover, animals from Ilemela district (*p*-value < 0.001) were significantly more colonized than animals from other districts (*p*-value < 0.001; **Table 2**). Animals with previous history of non-beta-lactam antibiotics use were less likely to carry ESBL as compared to those with no history of non-beta-lactam antibiotics use [11.9% (13/109) vs. 23.8% (117/491), *p*-value = 0.006]. Eight of the 109 animals with a history of antibiotic use (7.3%) were treated with sulfadimidine, 23 (21.1%) with penstreptomycine, 63 (58%) with oxytetracycline, and 15 (14%) with thiopurine.

**Table 2:** Association between ESBL carriage with predictor variables.

Variables		ESBL Carriage Status (*n* = 600)		*p*-value
			
		YES (*n* = 130) *n* (%)	NO (*n* = 470) *n* (%)	
Sex	Female	77 (18.1)	349 (81.9)	0.001
	Male	53 (30.5)	121 (69.5)	
District	Nyamagana	20 (15.6)	108 (84.4)	<0.001
	Ilemela	107 (28.3)	272 (71.8)	
	Misungwi	3 (3.2)	90 (96.8)	
Animal	Dogs	51 (39.2)	49 (10.4)	<0.001
	Chickens	16 (12.3)	84 (17.9)	
	Pigs	43 (33.1)	57 (12.1)	
	Cattle	14 (10.8)	86 (18.3)	
	Sheep	3 (2.3)	97 (20.6)	
	Goats	3 (2.3)	97 (20.6)	
Breed	Local	73 (18.3)	327 (81.6)	0.004
	Exotic	57 (28.5)	143 (71.5)	
History of antibiotic use	No	117 (23.8)	374 (76.2)	0.006


On multivariate logistic regression analysis, exotic breed type [OR (95% CI) = 2.372 (1.460–3.854), *p*-value < 0.001] was found to be a predictor of ESBL carriage among animals (**Table 3**).

**Table 3:** Multivariate logistic regression analysis for predictor variables associated with ESBL carriage.

Predictor variable	OR	95% CI	*p*-value
Sex	1.171	0.727–1.886	0.518
District	1.255	0.762–2.067	0.373
Animal	0.478	0.395–0.579	<0.001
Exotic breed	2.372	1.460–3.854	<0.001
History of antibiotic use	0.224	0.114–0.437	<0.001


### Phylogenetic Analysis of the Sequenced Isolates

Within the sequenced *E. coli* isolates four phylogenetic groups were detected. Phylogenetic group A was present in 17, B1 in five, B2 in two, and D in a single isolate. Fourteen different sequence types (ST) were detected (**Supplementary Table S3**). The most prevalent ST was ST617 (7/25), followed by ST1303 (3/25), ST2852 (3/25), and ST131 (2/25). *E. coli* ST617 was present in four different species (cattle, chicken, dog, pig). *E. coli* ST2852 and ST131 were present both in pigs and dogs, whereas *E. coli* ST1303 was present in cattle and a pig. *E. coli* ST617 and ST44 are members of the same clonal complex (CC) ST10, therefore this CC is predominant in this study.

**FIGURE 1:**
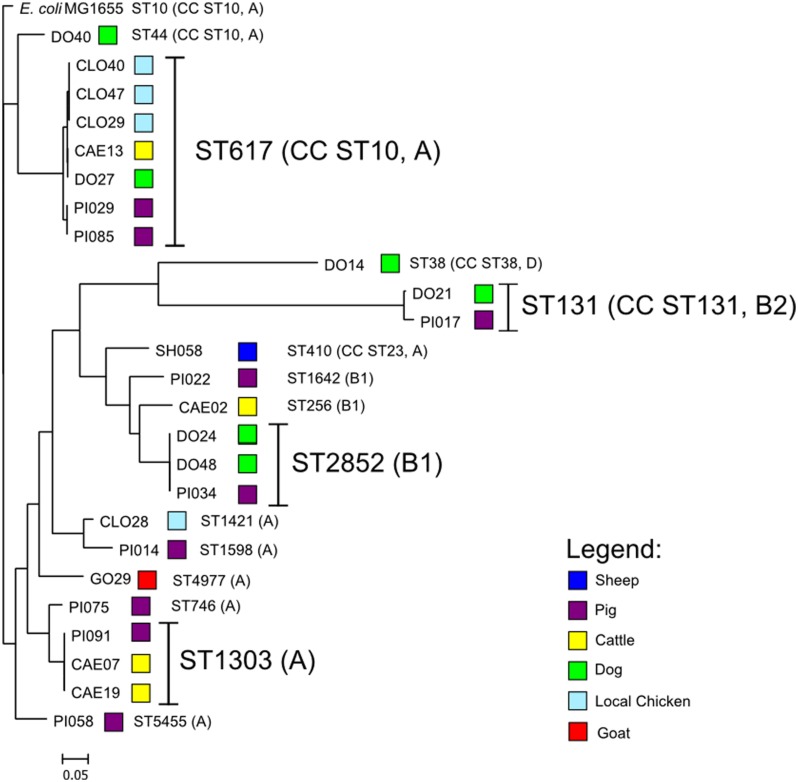
**Depiction of the core-genome based phylogenetic tree of the ESBL-producing *Escherichia coli* rooted with *E. coli* MG1655 genome using the Harvest Suite and MEGA5 software**.

Whole-genome phylogenetic analysis grouped the *E. coli* into two major clusters, one comprising only CC ST10 isolates and a second more diverse cluster with ST1303, ST2852, ST131, and other STs (**Figure 1**). STs that were present in more than one animal species were very closely related suggesting interspecies transfer of ESBL isolates.

### ESBL and Antibiotic Resistance Gene Alleles

All sequenced *E. coli* isolates harbored the *bla*_CTX-M-15_ allele and carried up to two additional non-ESBL beta-lactamases. A total of 60% (15/25) isolates harbored only *bla*_TEM-1B_ and two isolates (8%) carried only *bla*_OXA-1_. All other isolates displayed both *bla*_OXA-1_ and *bla*_TEM-1B_ (32%, 8/25). A total of eight different aminoglycoside resistance genes were detected. All isolates harbored both *strA* and *strB* excepting CAE02, DO14 (no *strA/strB*), and PI017 (only *strA*). Other aminoglycoside resistance genes included *aac(3)-IId* (6/25), *aac(3)-IIa (5/25), aadA5* (9/25)*, aadA1* (1/25), *aadA2* (1/25), and *aacA4* (1/25). Two different quinolone resistance genes were present: *aac(6′)-Ib-cr* (12/25 isolates), and *qnrS1* (11/25). Most of the isolates had either *sul1* or *sul2*, *dfrA14* or *dfrA17* and *tet(A)* or *tet(B)* genes conferring resistance to sulphonamides, trimethoprim or tetracycline (**Supplementary Table S3**).

### Location of *bla*_CTX-M-15_, Plasmid Replicons, and Plasmid Similarity

The *bla*_CTX-M-15_ allele was present in a plasmid-like environment in 21/25 (84%) isolates (**Supplementary Table S4**). Four isolates harbored a chromosomally inserted *bla*_CTX-M-15_ into three different hypothetical proteins (DO21, PI017, PI075) or into a type III secretion system (CAE02). Plasmid replicon types detected were IncFIA, IncFIB, IncFII, and IncY (**Supplementary Table S3**). pMLST typing of the IncF plasmids revealed the presence of the common plasmid type F31:A4:B1 (*n* = 4) and F31:A6:B1 (*n* = 4).

**FIGURE 2:**
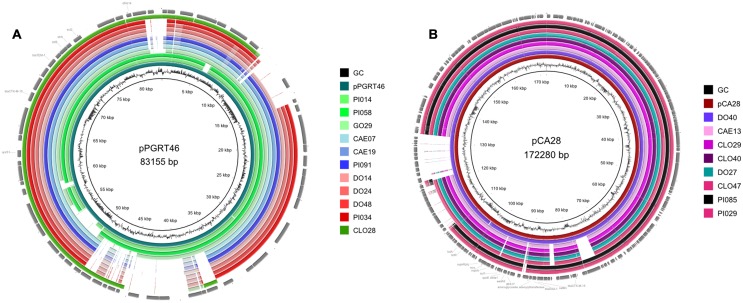
**Comparative analysis of the whole genome sequences with plasmid pPGRT46 **(A)** and plasmid pCA28 **(B)** using BRIG software**.

Further analysis indicated that 11/25 (44%) isolates carry a multi-resistance region similar to that present in the plasmid pPGRT46 (accession no. KM023153), isolated from healthy pregnant women in Nigeria, harboring *bla*_CTX-M-15_ and the quinolone resistance protein QnrS1 (Fortini et al., 2015). Bioinformatic analysis revealed that these plasmids were highly related to pPGRT46 (accession no. CP009232) with an overall homology of 64 to 95% (**Figure 2A**). Eight plasmids where highly homologous to the plasmid pCA28, isolated in the USA (Li et al., 2015) and displayed an overlap of 86 to 99% (**Figure 2B**). Four isolates harbored a resistance cassette that was identical to the one previously described in pSTm-A54650 (accession no. LK056646).

## Discussion

The present study indicates high fecal colonization of companion and domestic farm animals with ESBL-producing Enterobacteriaceae, with dogs and pigs displaying the highest colonization rates. This study documents the situation present in rural farming communities in the Mwanza region and indicates circulation of related *E. coli* clones among different animal species.

In our study, similar to other studies, *E. coli* comprised the majority of ESBL-producing bacteria in companion and domestic farm animals (Madec et al., 2008; Ewers et al., 2012; Geser et al., 2012; Munang’andu et al., 2012). Over two thirds of *E. coli* ESBL-producing isolates in the present study were resistant to trimethoprim/sulphamethoxazole and tetracycline as compared to less than one third exhibiting resistance to ciprofloxacin and gentamicin. The wide-spread use of oxytetracycline (58%) among companion and domestic farm animals in this study and another similar study in Morogoro may account for this high resistance trend (Katakweba, 2014).

Despite the fact that animal type and history of non-beta-lactam antibiotics use were protective following multivariate analysis, exotic breed animals were 2.4 times more likely to carry ESBL-producing isolates as compared to local breed animals. This may be related to excessive use of antimicrobial agents in exotic breeds as compared to local breeds, thereby increasing the selective pressure, mutation rates and promoting evolution of resistance (Bester and Essack, 2008).

To identify, whether there are common clones or plasmids in humans and animals in Tanzania, we compared the results of our study with earlier studies on human’s isolates. Firstly, isolates from humans and animals share antibiotic resistance patterns, i.e., to gentamycin, tetracycline, and trimethoprim/sulfamethoxazole (Kayange et al., 2010). Secondly, all animal isolates examined in this study harbored the *bla*_CTX-M-15_ allele, which is also present in the majority of *E. coli* isolates circulating in the hospital in the same region (Mshana et al., 2011b; Katakweba, 2014). There is only a small overlap between clonal populations of *E. coli* present in either population. Thus it is likely that this distribution may be related to the presence of commonly occurring plasmids, rather than clonal isolates. This is in keeping with results obtained in a previous study (Mshana et al., 2011b), conducted in the same region, which observed that ST131 (CC ST131) of phylogroup B2 was the most common ST obtained from human clinical isolates. The majority of clones observed here, i.e., CC ST10 (ST617, ST44) of phylogroup A, is distinct from that seen in the human isolates. The presence of ST131 isolates in this study suggests close contact between humans and animals.

The predominance of *E. coli* ST617 suggests clonal distribution between different animal species. Eight (32%) of the sequenced isolates were members of the clonal complex CC ST10. The presence of CC ST10 ESBL-producing *E. coli* in humans has been commonly reported in Africa (Fam et al., 2011; Aibinu et al., 2012; Fortini et al., 2015; Rafaï et al., 2015; Sallem et al., 2015). In all countries these strains harbor *bla*_CTX-M-15_ genes in multiple IncF plasmids, but are also associated with a variety of other plasmid replicons, including rare plasmid types such as IncY and IncQ. In this study, eleven isolates were found to carry a plasmid with homology to the Inc-untypable plasmid pPGRT46 recently detected in Nigeria in *E. coli* isolates from humans. This indicates that homologous plasmids might have spread among different *E. coli* strains in the continent not only between humans, but also in animals. Moreover, these plasmids harbor adjacent *bla*_CTX-M-15_ and *qnrS1* resistance genes and thereby confer both ESBL- and quinolone resistance resulting in a co-transfer of these resistances in the worst case, even if only one of these two antibiotic classes is used. Eight isolates carried a plasmid with homology to the pCA28 (F31:A4:B1) plasmid which has been recently found in the USA (Li et al., 2015). Interestingly, one canine isolate (DO40) harbored a plasmid that was not only 99% similar to the plasmid pCA28, but also had the same ST as the *E. coli* isolate, from which pCA28 was originally isolated (ST44). We note that CTX-M-15 was also present as chromosomal insertions of the *bla*_CTX-M-15_ allele in diverse strains of *E. coli*.

As in a previous study (Mshana et al., 2011b) conducted in the same region the majority of *E. coli* isolates had IncF plasmids with additional transferable resistances being gentamycin, tetracycline and trimethoprim/sulfamethoxazole. Unlike the previous study where no IncY plasmids were detected, isolates from animals 8/25 (32%) harbor IncY plasmids carrying *bla*_CTX-M-15_, *bla*_TEM-1B_, *strA*, *strB*, and *qnrS1.*

## Conclusion

This study for the first time conducted in Tanzania examines the presence of CTX-M-15-producing *E. coli* in rural farming communities in Mwanza, Tanzania. Among these isolates, the CC ST10 (ST617, ST44) was predominant. The transmission of *bla*_CTX-M-15_ was probably plasmid driven and involved plasmid populations that are regionally present in East and West Africa. The high fecal carriage of ESBL-producing Enterobacteriaceae among companion and domestic farm animals harboring predominantly *bla*_CTX-M-15_ in combination with quinolone and aminoglycoside resistance genes underscores the need of an integrated antimicrobial surveillance system in veterinary and human medicine to investigate the potential sources and the dynamics of transmission.

## Author Contributions

JS, MMM, MR, CI, TC, and SM conceived, designed and executed the study; JS, NS, and MMM collected the data and samples; JS, NS, and SM performed laboratory analysis; LF and TC performed WGS; JS, LF, NS, MMM, TC, and SM analyzed the data; JS, LF, TC, and SM wrote the manuscript which was critically reviewed by all authors. All authors have read and approved the final draft of the manuscript.

## Conflict of Interest Statement

The authors declare that the research was conducted in the absence of any commercial or financial relationships that could be construed as a potential conflict of interest.
