# Supplementary material for: Multiple ESBL-Producing Escherichia coli Sequence Types Carrying Quinolone and Aminoglycoside Resistance Genes Circulating in Companion and Domestic Farm Animals in Mwanza, Tanzania, Harbor Commonly Occurring Plasmids
Source: Front Microbiol. 2016 Feb 11;7:142. doi: 10.3389/fmicb.2016.00142 (PMC4749707; doi:10.3389/fmicb.2016.00142)
Supplement: Supplementary file 1 [file Table_1.DOCX]

**Supplementary Table 1: Baseline characteristics of companion and domestic farm animals**

| **Variable** | | **Amount (n)** | **Percentage (%)** |
| --- | --- | --- | --- |
| **Sex** | Female | 426 | 71.0 |
|  | Male | 174 | 29.0 |
| **District** | Nyamagana | 128 | 21.3 |
|  | Ilemela | 379 | 63.2 |
|  | Misungwi | 93 | 15.5 |
| **Breed type** | Local | 400 | 66.7 |
|  | Exotic | 200 | 33.3 |
| **History of antibiotic use** | No | 491 | 81.8 |
|  | Yes | 109 | 18.2 |
