# Supplementary material for: Multiple ESBL-Producing Escherichia coli Sequence Types Carrying Quinolone and Aminoglycoside Resistance Genes Circulating in Companion and Domestic Farm Animals in Mwanza, Tanzania, Harbor Commonly Occurring Plasmids
Source: Front Microbiol. 2016 Feb 11;7:142. doi: 10.3389/fmicb.2016.00142 (PMC4749707; doi:10.3389/fmicb.2016.00142)
Supplement: Supplementary file 2 [file Table_2.DOCX]

**Supplementary Table 2: Proportions of ESBL carriage among companion and domestic farm animals**

| **Animal type** | **Amount of ESBL carriage (n)** | **Percentage (%)** |
| --- | --- | --- |
| Dog | 51 | 39.2 |
| Cattle | 14 | 10.8 |
| Chicken | 16 | 12.3 |
| Goat | 3 | 2.3 |
| Pig | 43 | 33.1 |
| Sheep | 3 | 2.3 |
| **Total** | **130** | **100** |
