# Supplementary material for: Multiple ESBL-Producing Escherichia coli Sequence Types Carrying Quinolone and Aminoglycoside Resistance Genes Circulating in Companion and Domestic Farm Animals in Mwanza, Tanzania, Harbor Commonly Occurring Plasmids
Source: Front Microbiol. 2016 Feb 11;7:142. doi: 10.3389/fmicb.2016.00142 (PMC4749707; doi:10.3389/fmicb.2016.00142)
Supplement: Supplementary file 3 [file Table_3.DOCX]

**Supplementary Table 3: Characteristics of the 25 sequenced ESBL-producing *E. coli***

| **Strain** | **Antibiotic resistance^a^** | **MLST** | **Phylogroup** | **Beta-Lactam and PMQR genes** | **Plasmid Replicon type** | **pMLST of IncF plasmids** |
| --- | --- | --- | --- | --- | --- | --- |
| CAE02 | CIP, GEN, TET, SXT | ST256 | B1 | ^b^*bla*_CTX-M-15_, *bla*_TEM-1B_, *aacA4, aac(6’)-Ib-cr* | No replicon | - |
| CAE07 | CIP, GEN, TET, SXT | ST1303 | A | *bla*_CTX-M-15_, *bla*_TEM-1B_, *strA*, *strB*, *qnrS1* | Y | - |
| CAE13 | CIP, GEN, TET, SXT | ST617 | A | *bla*_CTX-M-15_, *bla*_OXA-1_, *bla*_TEM-1B_, *aadA5*, *aac(6’)-Ib-cr*, *aac(3)-IId, strA, strB* | FIA, FIB, FII | F31:A4:B1 |
| CAE019 | CIP, GEN, TET, SXT | ST1303 | A | *bla*_CTX-M-15_, *bla*_TEM-1B_, *strA*, *strB*, *qnrS1* | Y | - |
| CLO28 | CIP, GEN, TET, SXT | ST1421 | A | *bla*_CTX-M-15_, *bla*_TEM-1B_, *strA*, *strB*, *qnrS1* | No replicon |  |
| CLO29 | CIP, GEN, TET, SXT | ST617 | A | *bla*_CTX-M-15_, *bla*_OXA-1_, *bla*_TEM-1B_, *aadA5, aac(6’)-Ib-cr, aac(3)-IId*, *strA*, *strB* | FIA, FIB, FII | F31:A6:B1 |
| CLO040 | CIP, GEN, TET, SXT | ST617 | A | *bla*_CTX-M-15_, *bla*_OXA-1_, *bla*_TEM-1B_, *aadA5, aac(6’)-Ib-cr, aac(3)-IId*, *strA, strB* | FIB, FII | F31:A6:B1 |
| CLO047 | CIP, GEN, TET, SXT | ST617 | A | *bla*_CTX-M-15_, *bla*_OXA-1_, *bla*_TEM-1B_, *aadA5, aac(6’)-Ib-cr, aac(3)-IId*, *strA*, *strB* | FIA, FIB, FII | F31:A6:B1 |
| DO14 | CIP, GEN, TET, SXT | ST38 | D | *bla*_CTX-M-15_, *bla*_TEM-1B_, *strA*, *strB*, *qnrS1* | Y,B/O/K/Z, | - |
| DO21 | CIP, GEN, TET, SXT | ST131 | B2 | ^b^*bla*_CTX-M-15_, *bla*_OXA-1_, *aac(3)-IIa*, *aac(6’)-Ib-cr* | X1, FIB, FII | F36:A-:B1 |
| DO24 | CIP, GEN, TET, SXT | ST2852 | B1 | *bla*_CTX-M-15_, *bla*_TEM-1B_, *strA*, *strB*, *qnrS1* | Y | - |
| DO27 | CIP, GEN, TET, SXT | ST617 | A | *bla*_CTX-M-15_, *bla*_OXA-1_, *bla*_TEM-1B_, *aadA5*, *aac(6’)-Ib-cr, aac(3)-IId, strA*, *strB* | FIA, FIB, FII | F31:A6:B1 |
| DO40 | CIP, GEN, TET, SXT | ST44 | A | *bla*_CTX-M-15_, *bla*_OXA-1_, *aadA5, aac(6’)-Ib-cr, aac(3)-IIa, strA, strB* | FIB, FIA, FII | F31:A4:B1 |
| DO48 | CIP, GEN, TET, SXT | ST2852 | B1 | *bla*_CTX-M-15_, *bla*_TEM-1B_, *strA*, *strB*, *qnrS1* | Y | - |
| PI014 | CIP, GEN, TET, SXT | ST1598 | A | *bla*_CTX-M-15_, *bla*_TEM-1B_, *strA*, *strB*, *qnrS1* | FIB, Y | F-:A-:B30 |
| PI017 | CIP, GEN, TET, SXT | ST131 | B2 | ^b^*bla*_CTX-M-15_, *bla*_OXA-1_, *bla*_TEM-1B_, *aadA1, aadA2, aac(3)-IId, aac(6’)-Ib-cr, strA* | FIA, FIB, FII, Q1 | F1:A1:B20 |
| PI022 | CIP, GEN, TET, SXT | ST1642 | B1 | *bla*_CTX-M-15_, *bla*_TEM-1B_, *aac(6’)-Ib-cr*, *aac(3)-IIa*, *strA*, strB | FIA, FIB, X3, Q1 | F:A1:B1 |
| PI029 | CIP, GEN, TET, SXT | ST617 | A | *bla*_CTX-M-15_, *bla*_OXA-1_, *bla*_TEM-1B_, *aadA5, aac(6’)-Ib-cr, aac(3)-IIa, strA,* *strB* | FII, FIB, IFIA, X4 | F31:A4:B1 |
| PI034 | CIP, GEN, TET, SXT | ST2852 | B1 | *bla*_CTX-M-15_, *bla*_TEM-1B_, *strA*, *strB*, *qnrS1* | Y | - |
| PI058 | GEN, TET, SXT | ST5455 | A | *bla*_CTX-M-15_, *bla*_TEM-1B_, *strA*, *strB*, *qnrS1* | FIB | Unknown ST |
| PI075 | GEN, TET, SXT | ST746 | A | ^b^*bla*_CTX-M-15_, *bla*_TEM-1B_, *strA*, *strB* | FIB(K) | Unknown ST |
| PI085 | CIP, GEN, TET, SXT | ST617 | A | *bla*_CTX-M-15_, *bla*_OXA-1_, *bla*_TEM-1B_, *aadA5, aac(6’)-Ib-cr, aac(3)-IIa, strA, strB* | FII, FIB, FIA, X4 | F31:A4:B1 |
| PI091 | CIP, GEN, TET, SXT | ST1303 | A | *bla*_CTX-M-15_, *bla*_TEM-1B_, *strA*, *strB*, *qnrS1* | Y | - |
| SH058 | CIP, GEN, TET, SXT | ST410 | A | *bla*_CTX-M-15_, *bla*_OXA-1_, *bla*_TEM-1B_, *aadA5, aac(6’)-Ib-cr, strA, strB* | FIA, FIB, Q1 | F1:A1:49 |
| GO29 | GEN, TET, SXT | ST4977 | A | *bla*_CTX-M-15_, *bla*_TEM-1B_, *strA*, *strB, qnrS1* | FIB | Unknown ST |

^a^ abbreviation of resistance GM: Gentamicin, TET: Tetracycline, CIP: Ciprofloxacin, SXT: Sulphamethaxazole/trimethoprim ^b^in theses isolates, *bla*_CTX-M-15_ was present in a chromosomal location
