# Supplementary material for: Multiple ESBL-Producing Escherichia coli Sequence Types Carrying Quinolone and Aminoglycoside Resistance Genes Circulating in Companion and Domestic Farm Animals in Mwanza, Tanzania, Harbor Commonly Occurring Plasmids
Source: Front Microbiol. 2016 Feb 11;7:142. doi: 10.3389/fmicb.2016.00142 (PMC4749707; doi:10.3389/fmicb.2016.00142)
Supplement: Supplementary file 4 [file Table_4.DOCX]

**Supplementary Table 4: Analysis of the CTX-M-15 environment in the sequenced isolates**

| **Isolate** | **Location** | **Closest reference** | **Accession number of the reference** |
| --- | --- | --- | --- |
| CAE02 | genome | *Klebsiella pneumoniae* strain 6234 plasmid p6234-198.371kb | CP010390.1 |
| CAE07 | plasmid | *Escherichia coli* strain PGR46 plasmid pPGRT46 | KM023153.1 |
| CAE13 | plasmid | *Escherichia coli* strain CA28 plasmid pCA28 | CP009232.1 |
| CAE019 | plasmid | *Escherichia coli* strain PGR46 plasmid pPGRT46 | KM023153.1 |
| CLO28 | plasmid | *Escherichia coli* strain PGR46 plasmid pPGRT46 | KM023153.1 |
| CLO29 | plasmid | *Salmonella enterica* subsp. enterica serovar Typhimurium plasmid pSTm-A54650 | LK056646.1 |
| CLO040 | plasmid | *Salmonella enterica* subsp. enterica serovar Typhimurium plasmid pSTm-A54650 | LK056646.1 |
| CLO047 | plasmid | *Salmonella enterica* subsp. enterica serovar Typhimurium plasmid pSTm-A54650 | LK056646.1 |
| DO14 | plasmid | *Escherichia coli* strain PGR46 plasmid pPGRT46 | KM023153.1 |
| DO21 | genome | *Escherichia coli* plasmid pI1-34TF | LN850163.1 |
| DO24 | plasmid | *Escherichia coli* strain PGR46 plasmid pPGRT46 | KM023153.1 |
| DO27 | plasmid | *Escherichia coli* strain CA28 plasmid pCA28 | CP009232.1 |
| DO40 | plasmid | *Escherichia coli* strain CA28 plasmid pCA28 | CP009232.1 |
| DO48 | plasmid | *Escherichia coli* strain PGR46 plasmid pPGRT46 | KM023153.1 |
| PI014 | plasmid | *Escherichia coli* strain PGR46 plasmid pPGRT46 | KM023153.1 |
| PI017 | genome | *Shigella sonnei* strain SS084469 plasmid pSH4469 | KJ406378.1 |
| PI022 | plasmid | *Salmonella enterica* subsp. enterica serovar Typhimurium plasmid pSTm-A54650 | LK056646.1 |
| PI029 | plasmid | *Escherichia coli* strain CA28 plasmid pCA28 | CP009232.1 |
| PI034 | plasmid | *Escherichia coli* strain PGR46 plasmid pPGRT46 | KM023153.1 |
| PI058 | plasmid | *Escherichia coli* strain PGR46 plasmid pPGRT46 | KM023153.1 |
| PI075 | genome | *Escherichia coli* plasmid pI1-34TF | LN850163.1 |
| PI085 | plasmid | *Escherichia coli* strain CA28 plasmid pCA28 | CP009232.1 |
| PI091 | plasmid | *Escherichia coli* strain PGR46 plasmid pPGRT46 | KM023153.1 |
| SH058 | plasmid | *Klebsiella pneumoniae* subsp. pneumoniae strain 234-12 plasmid pKpn23412-362 | CP011314.1 |
| GO29 | plasmid | *Escherichia coli* strain PGR46 plasmid pPGRT46 | KM023153.1 |
